# Supplementary material for: Uncovering unseen fungal diversity from plant DNA banks
Source: PeerJ. 2017 Aug 28;5:e3730. doi: 10.7717/peerj.3730 (PMC5578370; doi:10.7717/peerj.3730)
Supplement: Table S1 [file peerj-05-3730-s001.docx]

| **Sample Code** | **HPDL Number** | ***Clermontia* species** | **Island** | **Date Extracted** | **Latitude** | **Longitude** | **Location** | **Collector** |
| --- | --- | --- | --- | --- | --- | --- | --- | --- |
| M1 | 6843 | *kakeana* | Moloka‘i | 7/14/11 | 21.13 | -156.92 | Kamakou Preserve | Richard Pender |
|  | 6844 |  |  |  |  |  |  |  |
| H1 | 6961 | *calophylla* | Hawai‘i | 11/18/11 | 20.09 | -155.74 | Pu‘u O ‘Umi Natural Area Reserve | Richard Pender |
|  | 6962 |  |  |  |  |  |  |  |
| H2 | 6888 | *kohalae* | Hawai‘i | 9/17/11 | 20.08 | -155.74 | Kohala Mts. | Richard Pender |
|  | 6889 |  |  |  |  |  |  |  |
| H3 | 6856 | *clermoniotides* | Hawai‘i | 8/3/11 | 19.21 | -155.60 | Ka‘u Preserve Kaiholena | Richard Pender |
|  | 6857 |  |  |  |  |  |  |  |
| H4 | 7339 | *peleana ssp. singulariflora* | Hawai‘i | 6/7/13 | 20.18 | -155.80 | Kohala Mts. | Richard Pender |
|  | 7940 |  |  |  |  |  |  |  |
| K1 | 5089 | *fauriei* | Kaua‘i | 9/6/05 | 22.09 | -159.59 | Alaka‘i Swamp | Clifford Morden |
|  | 5090 |  |  |  |  |  |  |  |
| O1 | 6809 | *kakeana* | O‘ahu | 6/17/11 | 21.34 | -157.82 | Mt. Tantalus, Ko‘olau Mts. | Richard Pender |
|  | 6810 |  |  |  |  |  |  |  |
| O2 | 7008 | *oblongifolia ssp. oblongifolia* | O‘ahu | 3/15/12 | 21.41 | -158.10 | Palikea, Waianae Mts. | Richard Pender |
|  | 7009 |  |  |  |  |  |  |  |
| Ma1 | 6875 | *arborescens* | Maui | 7/5/11 | 20.82 | -156.28 | Waihiei Makawao Forest Reserve | Hank Oppenheimer |
|  | 6876 |  |  |  |  |  |  |  |
| Ma2 | 6831 | *kakeana* | Maui | 7/14/11 | 20.80 | -156.23 | Makawao Forest Reserve | Richard Pender |
|  | 6832 |  |  |  |  |  |  |  |
