# Supplementary material for: Uncovering unseen fungal diversity from plant DNA banks
Source: PeerJ. 2017 Aug 28;5:e3730. doi: 10.7717/peerj.3730 (PMC5578370; doi:10.7717/peerj.3730)
Supplement: Table S2 [file peerj-05-3730-s002.docx]

|  | **Amplicon PCR** | |  | **Index PCR** |
| --- | --- | --- | --- | --- |
| Reagent | Concentration | Volume/rxn |  | Volume/rxn |
| DNA | Full | 1.0 |  | 1.0 |
| H2O | - | 10.3 |  | 23.0 |
| Q5 Mastermix | 2x | 12.5 |  | 25.0 |
| Forward Primer | 10μM | 0.6 |  | 0.5 |
| Reverse Primer | 10μM | 0.6 |  | 0.5 |
|  | PCR Volume | 25.0 |  | 50.0 |
|  | Temperature (°C) | Time (seconds) | | Time (seconds) |
| Initial Denature | 98 | 120 |  | 120 |
| Denature | 98 | 10 |  | 15 |
| Annealing | 51/54 | 10 |  | 15 |
| Extension | 72 | 15 |  | 25 |
| Final Extension | 72 | 120 |  | 120 |
|  | PCR Cycles | 22 |  | 22 |
|  |  |  |  |  |
