# Supplementary figures and images for: Uncovering unseen fungal diversity from plant DNA banks
Source: PeerJ. 2017 Aug 28;5:e3730. doi: 10.7717/peerj.3730 (PMC5578370; doi:10.7717/peerj.3730)

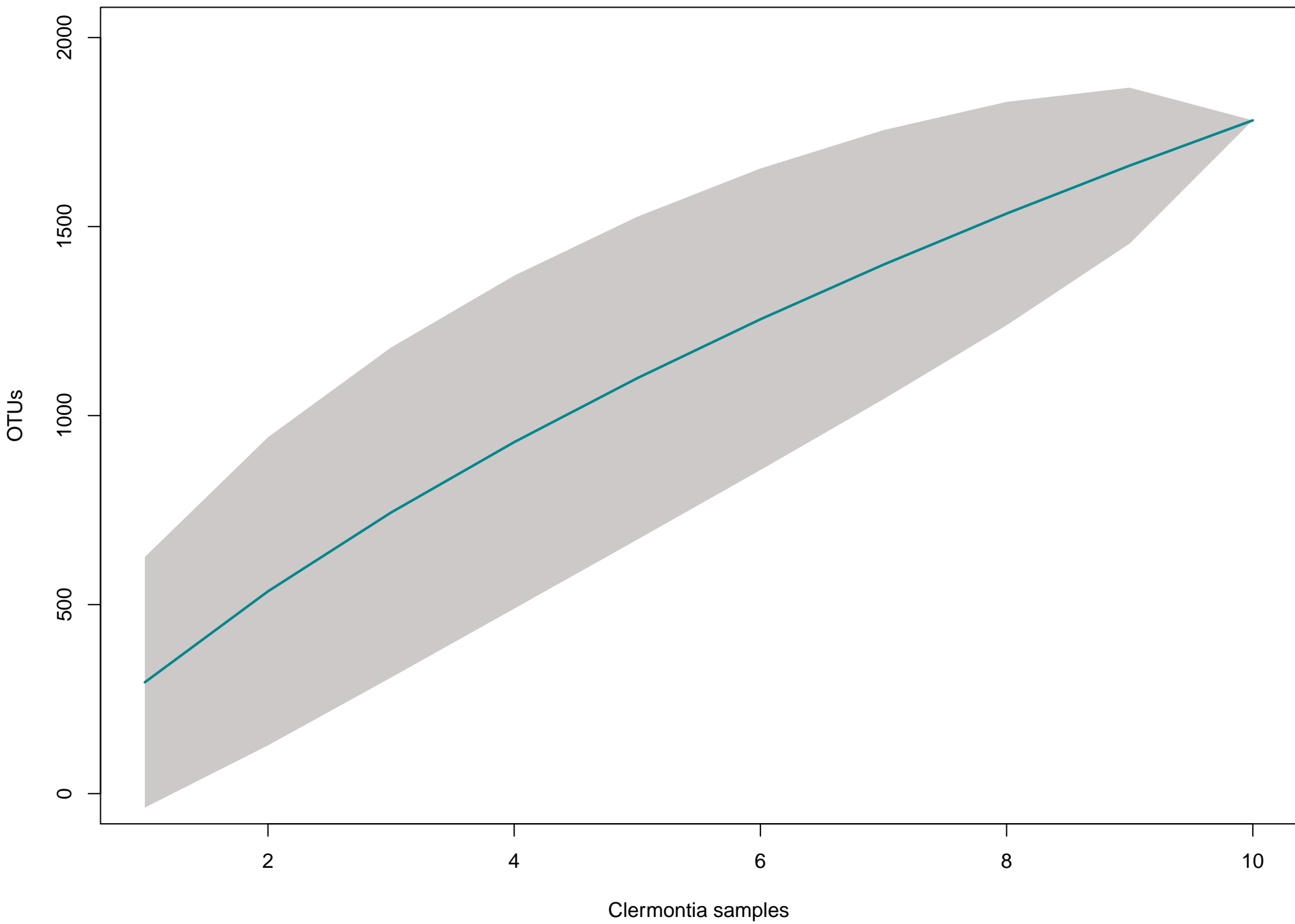

Supplement: Figure S1 [file peerj-05-3730-s003.pdf]

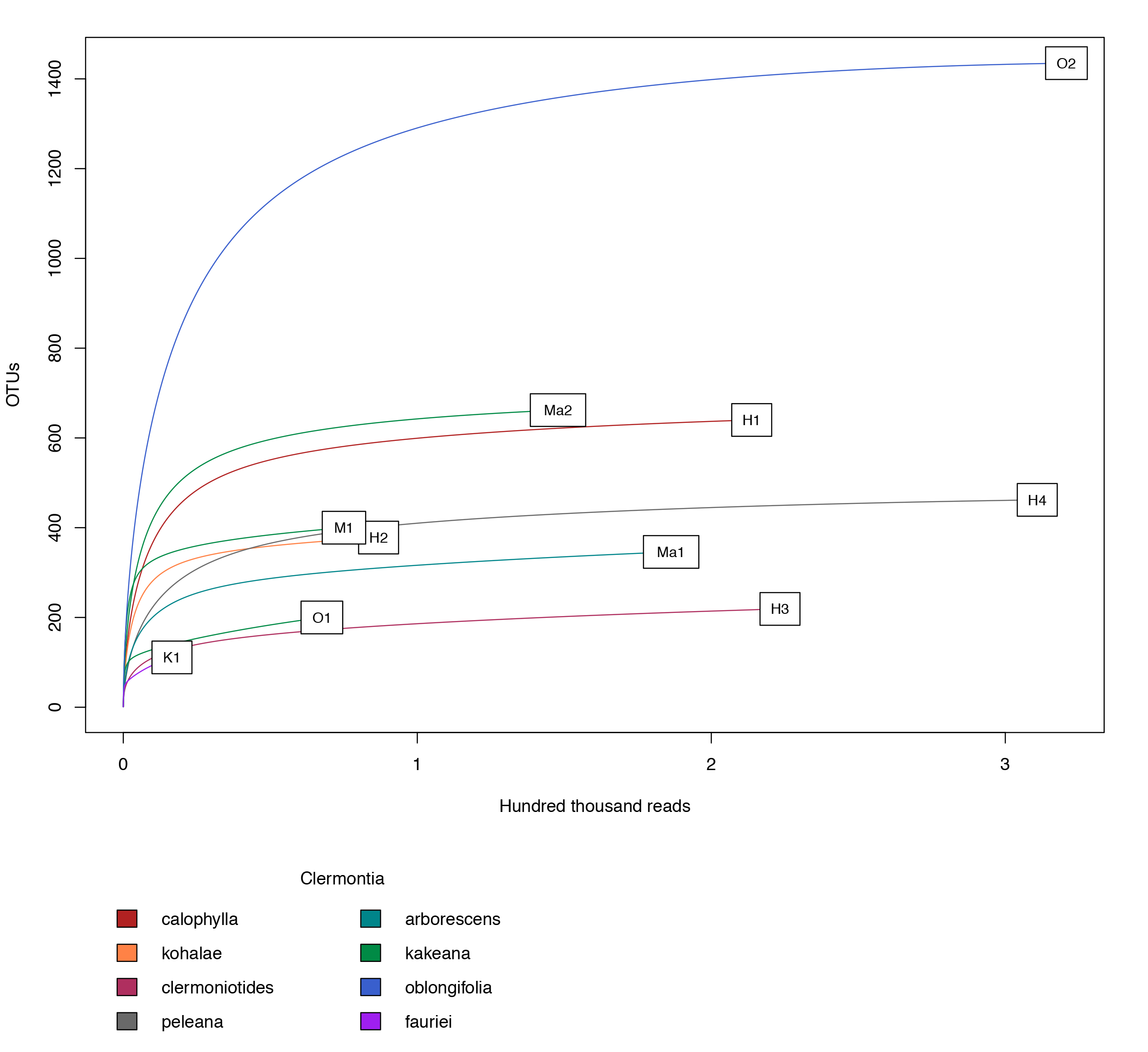

Supplement: Figure S2 [file peerj-05-3730-s004.png]

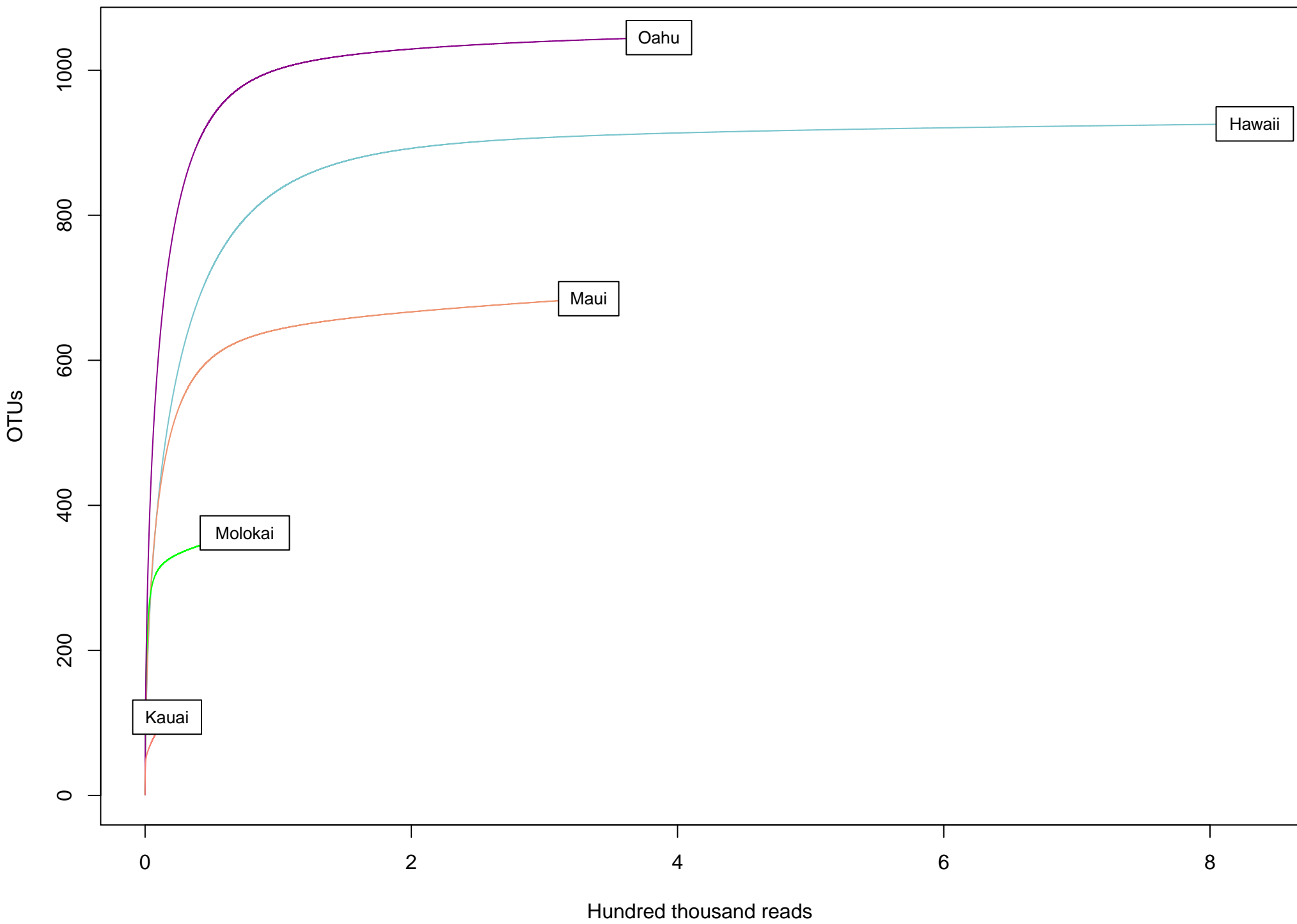

Supplement: Figure S3 [file peerj-05-3730-s005.pdf]

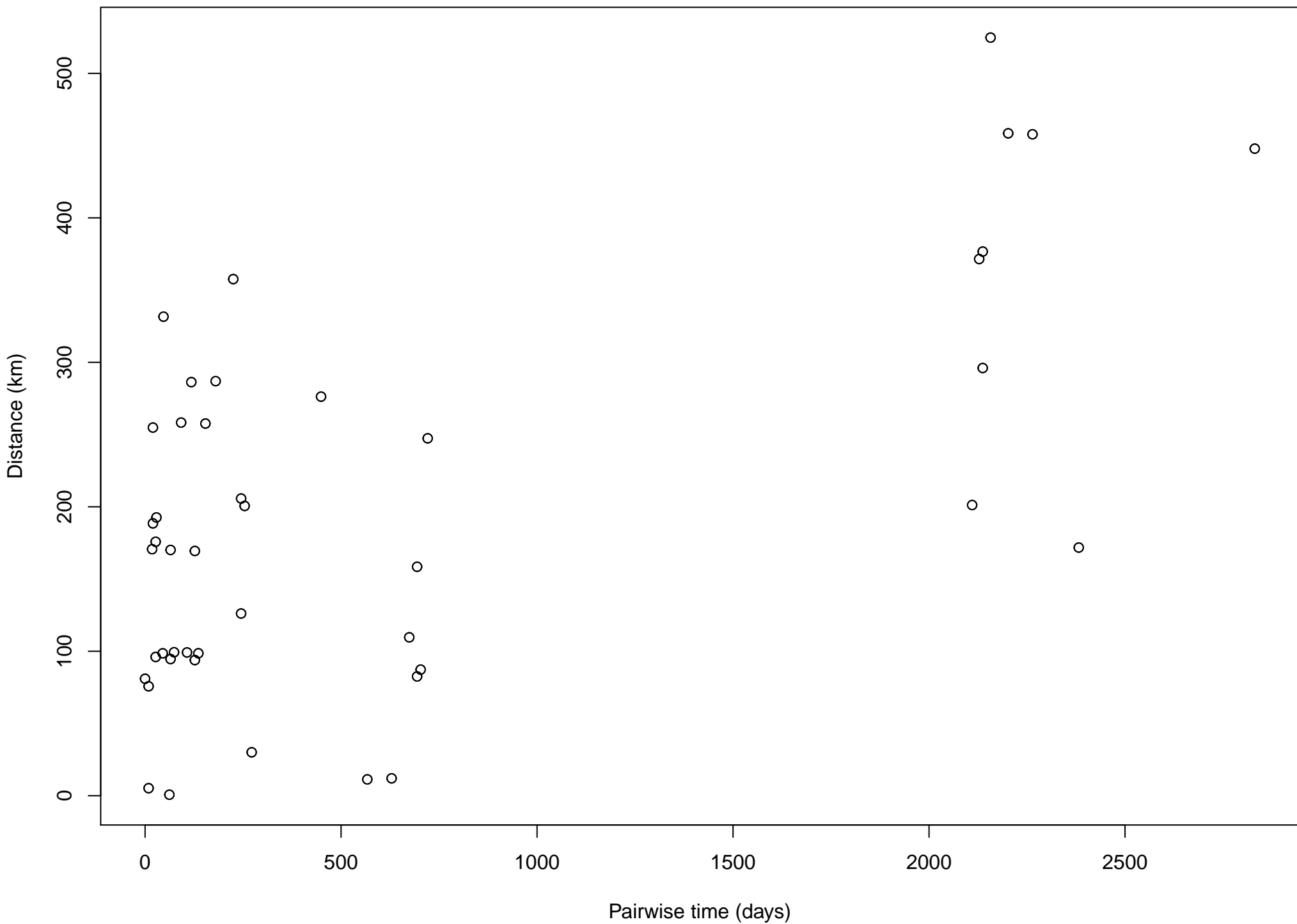

Supplement: Figure S4 [file peerj-05-3730-s006.pdf]

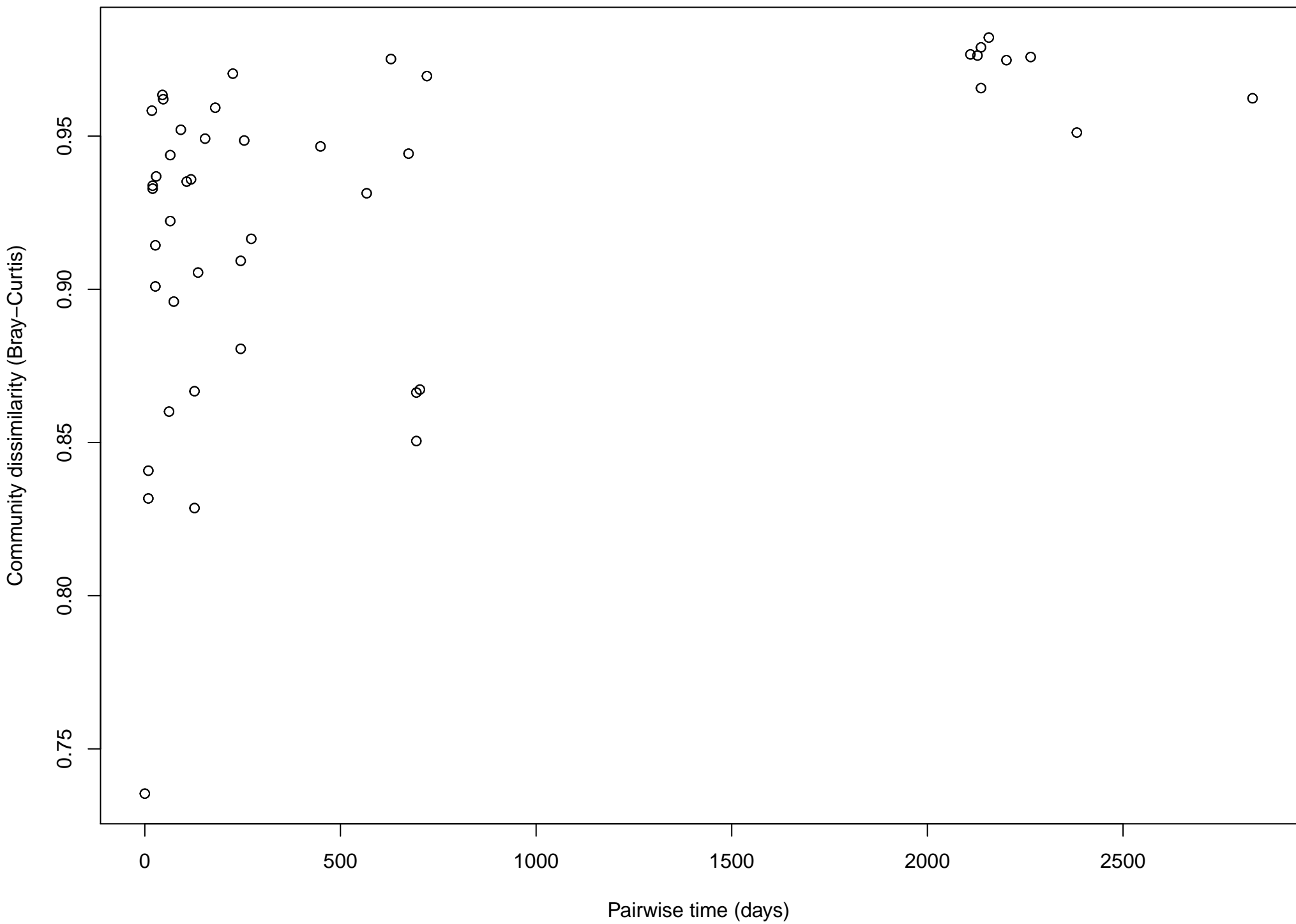

Supplement: Figure S5 [file peerj-05-3730-s007.pdf]

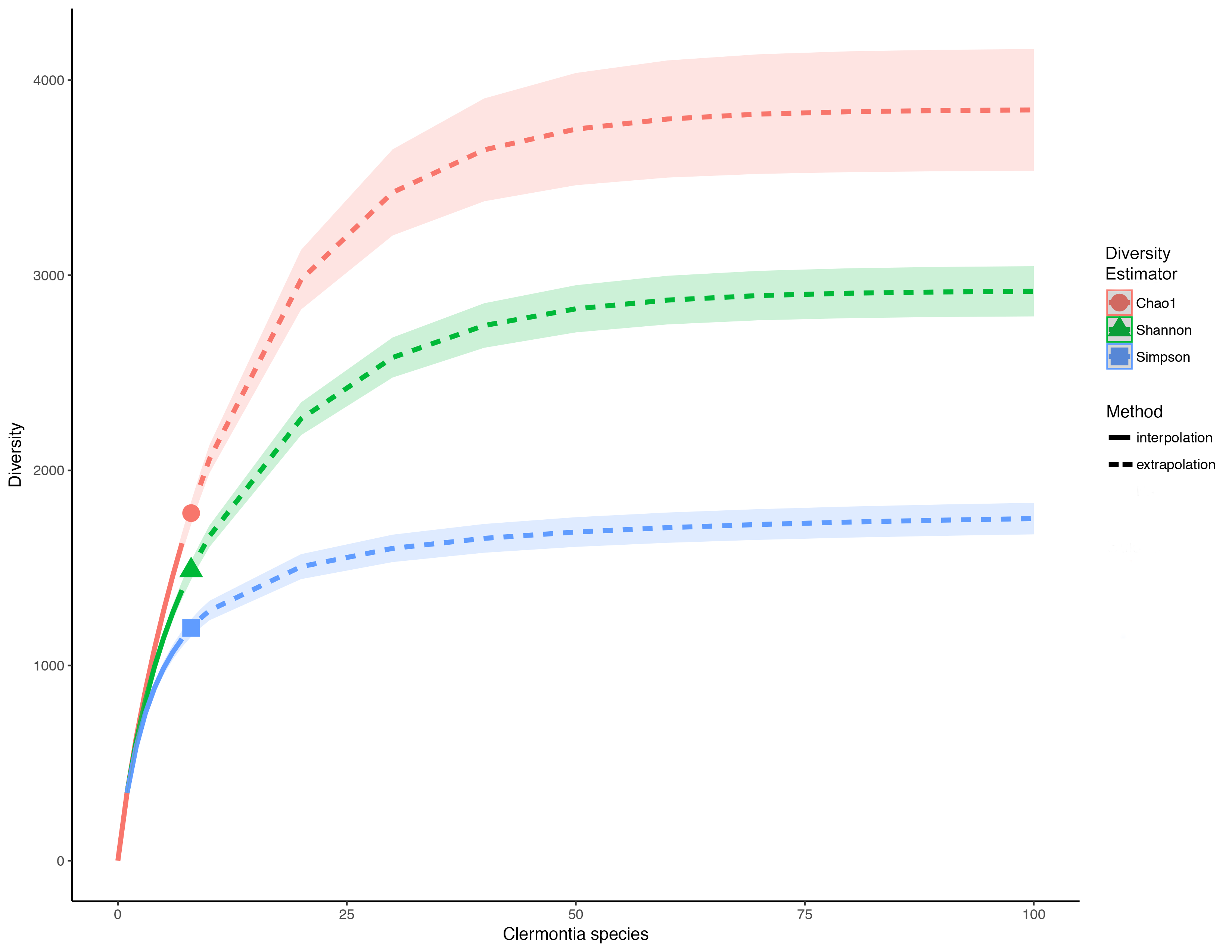

Supplement: Figure S6 — Based Hill’s numbers three different diversity estimators were used (Chao1 richness, exponential of Shannon entropy, and inverse Simpson concentration indices) and are shown by the different colors with 95% confidence intervals shown by shading. Shapes represent observed phylloplane fungal OTU diversity for eight species of Clermontia. [file peerj-05-3730-s008.png]
